# Dipsopathia—the Latest Medical Humbug

**Published:** 1844-02

**Authors:** 


*Dipsopathia,—the latest Medical Humbug.*—Extremes meet. The Medico-Chirurgical Review, for Jan. 1844, furnishes the following intelligence:

"Priessnitz has got a rival close to his own castle! About four miles from Graffenberg, in the lovely valley of Lindiviesse, Dr. Schrott, an old school-fellow of the apostle of hydropathy, has started a *cure-all*, in opposition to the Silesian peasant. His methodus medendi is no vile imitation of that of the far-famed practitioner of Graeffenberg. It is a veritable antipous. The one inculcates the drinking of water, and water only. Schrott forbids water and all other fluids—and thus cures by thirst!"

